# African Swine Fever (ASF): A Study to Identify Risk Factors Associated with the Introduction of the Disease into Pig Farms

**DOI:** 10.3390/pathogens15060569

**Published:** 2026-05-26

**Authors:** Silvia Bellini, Alessandra Scaburri, Matteo Tonni, Valentina Maggiano, Sara Fusar Poli, Martina Bernardis, Giovanni Santucci, Giovanni Loris Alborali

**Affiliations:** Istituto Zooprofilattico Sperimentale della Lombardia ed Emilia-Romagna, Via A. Bianchi 9, 25124 Brescia, Italy

**Keywords:** African swine fever, domestic pigs, wild boar, case–control study, risk factors, biosecurity measures

## Abstract

In 2023, ASF was recognized in Lombardy in wild boars, but shortly thereafter the disease was also identified in pig farms, with serious economic repercussions for the entire national pig sector. To identify factors related to the introduction of the infection into pig farms, a case–control study was conducted with the aim of gaining knowledge on the risk and protective factors involved in the introduction of the ASF virus into intensive pig farms. To this end, a questionnaire was developed on risk factors related to ASFV transmission into pig farms and on good management and biosecurity practices. The results of the study showed that measures aimed at strengthening the segregation of the farm from the external environment (external biosecurity), such as the mandatory passage through a hygiene lock upon entry, the presence of a hygiene lock for farm personnel and external visitors, the presence of special equipment and disinfection points at the entrance to the sheds, the loading of dead pigs outside the animal housing area and the ownership of non-adjacent agricultural land, were associated with a reduced risk. This information, if effectively communicated, could be of direct practical value to farmers to ensure the successful implementation of farm biosecurity.

## 1. Introduction

African swine fever (ASF) is a highly contagious viral disease that can be fatal for domestic pigs and wild boar. The disease is caused by a large DNA virus of the Asfarviridae family highly resistant in the environment [1]. The virus is mainly transmitted by direct contact between infected and susceptible pigs or through the ingestion of ASFV-contaminated pork by susceptible suids. The illegal movement of live pigs and pork is considered to be important for the long-distance spread of ASFV [2]. Other disease transmission pathways include vehicles and other fomites, such as clothing, footwear, surgical equipment, workers and visitors, slurries, and genetic materials. Therefore, human behaviour can play an important role in spreading the disease if adequate measures are not taken [3].

In certain areas, soft ticks of the genus Ornithodoros play a role, as biological vector, in transmitting the disease. However, their role in spreading the infection in the European countries in which ASF is currently found seems unlikely [4].

ASF has traditionally been present in the African continent. In 2007, genotype II of the ASFV was transported from Southeast Africa to Georgia [5] and has since gradually spread to neighbouring countries, affecting domestic and wild pigs [6]. Since then, outbreaks have occurred in Russia, Europe, China, Southeast Asia, and the Caribbean [7]. The first occurrence of ASF genotype II in the European Union (EU) was reported in 2014, and numerous EU countries have to date been affected by this devastating pig disease, which continues to spread.

In Italy, the first occurrence of ASFV genotype II was reported in January 2022, when the virus was identified in two wild boars found dead on the border between two regions in northwest Italy (Piedmont and Liguria). Since then, ASF has gradually spread in wild boar in the northwestern Italian regions and on 21 June 2023, a wild boar was found positive for ASFV in Lombardy, in a municipality in the province of Pavia, on the border with Piedmont. Two months later, the virus was detected in a pig farm (outbreak) in the same province. In total, between the end of August and September 2023, nine ASF outbreaks were identified in the province of Pavia, and they were eradicated in just over a month. Nevertheless, the infection continued to spread among wild boars and, in the following year, 21 further outbreaks were identified in the same area, all in commercial pig farms. Epidemiological investigations were carried out for all the outbreaks to identify and trace the source of infection as quickly as possible in order to break the cycle of ASFV transmission.

Previous studies conducted in some EU countries have reported that the specific routes of ASFV introduction into pig farms were identified in very few of the outbreaks for which detailed investigations were conducted. However, in the domestic pig sector, human activities are often considered the main driver of the spread of disease, despite the fact that the exact route of virus introduction into pig farms has rarely been detected [4]. In Lombardy, the epidemiological investigations carried out on the infected farms traced the movements of people (workers, veterinarians, technicians) and vehicles at risk, such as trucks transporting feed, animals, and carcasses [8]. However, the results of the investigations showed that, in some outbreaks during the risk period, several possible routes of infection were detected, and it was not always possible to identify which of these actually transmitted the infection. In contrast to what has been reported in other countries affected by ASF, wild boars may have played a role in the initial introduction of the infection into domestic pig farms, while the subsequent spread of the infection occurred through typical farm-to-farm transmission routes. In this regard, it is worth mentioning that, to date, in some areas of Italy affected by ASF in domestic pigs, the disease has never been identified in wild boars. In both epidemics, the infection was rapidly eradicated in domestic pigs, but persisted in wild boars, and this means it is a continued threat to domestic pigs. The fact that the virus is extremely resistant in the environment, remaining infectious for extended periods in matrices such as blood, meat, offal, soil, and fomites [9], also makes it a territorial risk.

To identify risk factors associated with the spread of ASFV, a case–control study was conducted on the risk and protective factors involved in its introduction into pig farms, as this is of direct practical value for farmers.

To this end, a specific questionnaire was developed based on the risk factors associated with the introduction of ASFV into different European pig farming systems [10] and a recent European Food Safety Authority (EFSA) publication reviewing the risk factors involved in the epidemiology of ASF [4]. Additionally, since the EFSA reported that inadequate biosecurity was likely to have contributed to the introduction of ASFV into domestic pig farms through indirect contact via contaminated fomites or the environment [11,12], the questionnaire also took into account the key preventive measures which are known to minimize the risk of the spread of ASFV in pig farming systems [13] and the biosecurity principles listed in Biocheck.UGent [14] and ClassyFarm.it [15].

## 2. Materials and Methods

### 2.1. Study Design and Farm Selection

To investigate the risk and protective factors associated with the introduction of ASFV into pig farms, a matched case–control study was implemented.

Two national information systems served as the primary sources of data. The National Animal Health Information System (SIMAN) was used to retrieve the complete list of ASF outbreaks in pig farms during the study period, including outbreak dates, type of farm, and geolocation. Complementary data on all pig farms operating in Lombardy, including details of their production category and geographical coordinates, were extracted from the National Livestock Database (BDN).

The case–control study focused on 21 ASF outbreaks detected between 25 July 2024, and 18 October 2024. During this period, the disease was already reported in wild boars, so the possibility of the disease spreading from wild boars to domestic pigs was investigated. To this end, the 17 wild boars that tested positive between 1 July and 31 October 2024, were included in the assessment.

For each outbreak, two control farms were selected using a matching procedure designed to minimize structural and geographical factors. Controls had to share the same type of farm as the corresponding case (fattening or breeding) and be located within a 20 km radius of the outbreak. Additionally, all control farms had to fulfil the following eligibility criteria: (i) being operational during the ASF epidemic; (ii) not been infected; and (iii) still housing pigs when the study was conducted. This matching strategy was adopted to ensure comparability between farms.

The study was conducted in the Lombardy area under restriction for ASF, in accordance with Annex I to Regulation (EU) 2023/594.

### 2.2. Questionnaire Development and Administration

An ad hoc questionnaire was developed based on the risk factors identified by the EFSA [4] and those reported by Bellini et al. [2], which also took into consideration the different pig farming systems. Moreover, specific questions on farm management practices were included considering the key biosecurity principles of the tools developed by Ghent University (Biocheck.UGent, biocheckgent.com) and included in the Italian ClassyFarm system (classyfarm.it).

The questionnaire contained questions and sub-questions, for a total of 69 items collecting information on the farm and personnel (30 questions), the presence of wild boar (4 questions), feed, drinking water and bedding (5 questions), farm management practices (5 questions), and biosecurity (25 questions).

The complete questionnaire is available in the Supplementary Material (Table S1).

The on-farm questionnaire was completed jointly by two veterinarians: an official veterinarian from the Local Health Authority (ATS) and a veterinarian from Istituto Zooprofilattico Sperimentale della Lombardia e dell’Emilia Romagna (IZSLER) with expertise in evaluating farm biosecurity and in ASF. The official veterinarians who, in accordance with a National Decree, are responsible for assessing the biosecurity of farms [16], visited each farm and completed the questionnaire regarding potential risk factors related to management and biosecurity measures. The risk period was considered to be twice the maximum ASF incubation period (fifteen days) (15 × 2 = 30 days) [17].

For the infected farms, the questionnaire was completed during the epidemiological investigation, whereas for control farms, the questionnaire was filled in at the end of the epidemic during a joint veterinary inspection on farm biosecurity. In fact, during the epidemic, it was considered dangerous to visit healthy farms located in high-risk areas and in proximity to infected farms.

### 2.3. Statistical Analysis

The results of the questionnaire were registered in a structured database used for data analysis.

The data were analyzed using conditional logistic regression models, in which disease status (case vs. control) was used as the outcome variable, while each questionnaire item was included as an explanatory variable.

To simplify the analysis, and due to the small number of farms, categorial variables were transformed into binary variables. The McNemar continuity correction was incorporated into the conditional logistic regression model to adjust for zero-cell counts in the paired contingency table, thereby improving the stability and validity of the parameter estimates under sparse data conditions.

Prior to conducting the analysis, the replies to some questions were coded to reflect exposure status. This approach improves the interpretability of the results. Responses were dichotomized, assigning a value of 1 to indicate exposure and 0 to indicate non-exposure. For example, Question 11 was coded as exposure = 1 when the response was “Introduction of pigs and/or piglets in the last 30 days”, and exposure = 0 when the response was “No introduction of pigs and/or piglets in the last 30 days”. Question 36-B lists the personnel who have visited the clean area of the farm in the last month. A new variable named Question 36-B aggregated was created. Grouping was performed to reduce the number of categories and to ensure a sufficient number of farms within the two groups. Afterwards, Question 36-B aggregated was coded as exposure = 1 if the response indicated “Internal and external personnel”, and exposure = 0 when the response referred exclusively to “Internal personnel”.

The results were considered statistically significant at 5% (*p* < 0.05).

### 2.4. Territorial Analysis

The local area of ASF outbreaks is known to influence the probability of the spread of the ASFV between pig farms and from wild boars to domestic pigs, and vice versa. ASFV spread has been positively correlated with spatial proximity between farms, which facilitates the spread of the infection over short distances and the possible formation of spatial clusters [18,19]. The geographical proximity to ASF-positive wild boars has been identified as one of the main determinants associated with infection in pig farms [20]. Based on this evidence, a local area analysis was conducted with the aim of understanding if the spatial distribution of positive cases could be correlated to the local spread of the infection. Descriptive statistics were computed to assess the distance between each outbreak, the nearest positive wild boar and the distance between different outbreaks.

Spatial and temporal information on ASF cases was obtained from SIMAN, which serves as the official Italian database for reports of positive cases of ASF in domestic pigs and wild boars. For each case, SIMAN records geographical coordinates, suspected date of infection, and confirmation status, enabling the reconstruction of the spatiotemporal sequence of the infection.

#### 2.4.1. Outbreak-to-Outbreak Pathway

To investigate outbreak-to-outbreak transmission, individual outbreaks were ordered chronologically according to the suspected date of infection.

For each outbreak, the distance to the subsequent outbreak was calculated, in order to assess both spatial proximity and temporal progression. Distances between outbreaks were summarized using descriptive statistics (median, IQR, minimum and maximum values). However, due to the matching criteria applied in the case–control study, the controls were selected based on proximity to the corresponding outbreak, as previously mentioned. Therefore, the distance between case and control could not be used for analyzing this pathway of transmission.

#### 2.4.2. Wild Boar-to-Outbreak Pathway

To investigate this transmission route, the minimum distances between the 17 positive wild boars detected between 1 July and 31 October 2024 and the 21 outbreaks were calculated. A temporal restriction was applied and only wild boars infected before the outbreaks in domestic pigs were considered in the analysis. This criterion ensured the correct chronological relationship between wild boars and outbreaks, avoiding incorrect associations with events that occurred after the outbreak.

The minimum distances obtained were summarized using descriptive statistics (minimum, maximum, quartiles and median), providing a description of the spatial characteristics between positive wild boars and domestic outbreaks.

To further characterize the spatial distribution of infected wild boars, a Kernel Density Estimation (KDE) map was generated using the geographical coordinates of ASF-positive wild boars. This map identified areas with a higher density of positive wild boars, which offered a visual indication of their proximity to the outbreaks.

### 2.5. Software

The statistical analysis was performed using software R, version 4.4.2 [21] and maps were produced using QGis software, version 3.34 [22].

## 3. Results

### 3.1. Case–Control Study: Survey Results

**Figure 1:**
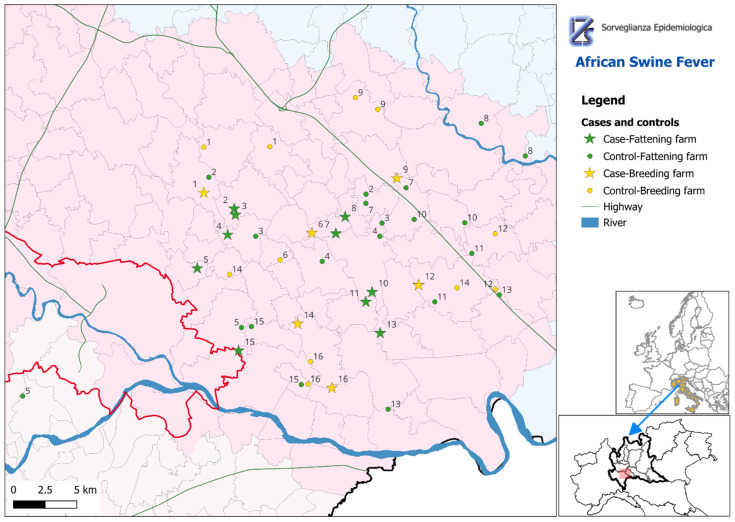
Map of ASF cases and controls with matching numbers (yellow = breeding farm; green = fattening farm).

The case–control analysis included 16 outbreak farms and 32 matched control farms. Five of the 21 outbreaks could not be paired with a control: in three cases because there was no eligible control farm located within the 20 km radius and in another two cases because there was no farm of the same category within the matching distance. In total, 48 pig farms were included in the dataset (Figure 1), and the results were summarized using descriptive statistics (Table S2).

There was no difference in the distribution of the number of pigs between cases and controls (Approximative Wilcoxon–Mann–Whitney test, *p* = 0.247) for both fattening (*p* = 0.235) and breeding (*p* = 0.665) farms.

### 3.2. Statistical Analysis

The analysis was carried out using conditional logistic regression models, with disease status (case or control farms) as the outcome variable.

Prior to modelling, collinearity among covariates was assessed using variance inflation factor (VIF) analysis. The VIF values were systematically extremely high and, in several cases, undefined, indicating perfect or near-perfect collinearity. Given that perfect collinearity violates the core assumptions of multivariable regression, multivariable modelling was deemed unfit for this study. Consequently, the analytical strategy relied on fitting separate univariable models to examine the association between each covariate and the outcome, with each model including a single covariate as an explanatory variable.

The results identified several variables significantly associated with the risk of ASF infection (Table 1). In particular, the presence of a house on the farm premises showed a strong association with increased risk. Other factors positively associated with risk included contact by the owner, family members, or employees with other pig farms, the presence of agricultural vehicles inside the clean area of the farm, and specific manure transfer practices involving internal vehicles. Additionally, it was found that visits to the clean area of the farm by external personnel (photovoltaic panel installation and fence repair) increased the risk, with an odds ratio of 27.00 (95% CI: 1.60–454.21). McNemar’s correction was applied because none of the control farms were visited by external personnel in the risk period.

Some biosecurity measures were significantly associated with a reduced risk of ASF infection (Table 2). These included the mandatory passage through a hygiene lock upon entry, the presence of disinfection points at the entrance to the sheds and loading of dead pigs outside the animal housing and management area (performed by the rendering company), and the ownership of non-adjacent agricultural land.

In addition, when McNemar’s correction was applied, the two variables “availability of a hygiene lock for farm personnel and external visitors”, and “presence of proper equipment” were identified as protective factors against ASFV introduction, with odds ratios of 0.09 (95% CI: 0.03–0.311) for the presence of a hygiene lock and 0.22 (95% CI: 0.10–0.50) for proper equipment. Furthermore, imposing mandatory passage through the hygiene lock upon entry was also associated with a reduced risk.

The farms included in the study were limited but these results of the analysis showed a statistical association between the investigated covariates and the introduction of the virus into the farm. Given the small number of cases and controls, non-statistically significant results cannot be interpreted as evidence of the absence of an association. These results should be interpreted with caution.

### 3.3. Territorial Analysis

Considering the criteria adopted for the case–control study, the distance between farms was unsuitable for the analysis of outbreak-to-outbreak transmission, since the controls were selected using proximity to the outbreak as one of the matching criteria, as described. However, in a previous study [8] of the same ASF epidemic, it was shown that the outbreaks that occurred in the area that had not been affected in 2023 were clustered and occurred in the protection zone (3 km radius) of the previous outbreaks and those beyond 3 km were found to be epidemiologically connected to those within the protection zone. These results are similar to those described in EFSA studies, where it was observed that outbreaks had an average distance from the nearest outbreak of 3.8 km [4,20]. Clustering was not observed in the first five outbreaks of 2024; these outbreaks occurred in an area also affected by ASF in 2023, where pig density was very low, with a median number of pigs per km^2^ of 21.

For the assessment of wild boar-to-outbreak transmission, a preliminary analysis was performed on all the outbreaks, and the distance between each wild boar and the nearest outbreak was calculated. Since distinct epidemiological conditions coexisted in the study area, in relation to the presence or absence of the disease in wild boars and also in relation to the density of pigs per km^2^, a separate evaluation was performed for the five outbreaks that occurred within the areas affected in 2023 and 2024, those excluded from the case–control analysis and the remaining 16 outbreaks.

The results of the analysis are summarized in Table 3, which reports the descriptive statistics of the minimum distances calculated.

**Figure 2:**
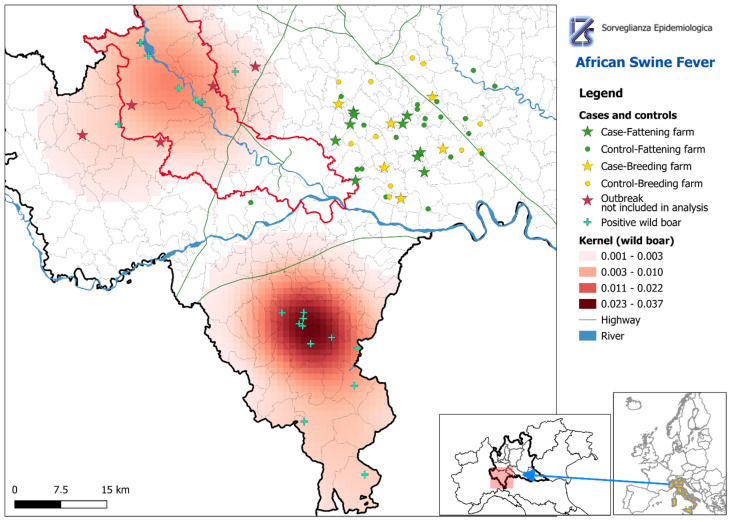
Kernel density map of positive wild boars detected during the period of the study.

As shown in Table 3, the five outbreaks excluded from case–control analysis occurred in an area closer to positive wild boars, with minimum and median distances of 3.53 km and 9.01 km, respectively. By contrast, the 16 outbreaks included in the study occurred in an area where no positive wild boars had been previously detected; the minimum and median distances observed were 22.24 km and 28.51 km, respectively. Quartile values also indicate that the outbreaks included in the case–control study were consistently further away from positive wild boars than those excluded. In the previous study, which described the epidemiological characteristics of the disease in the same area, farm-to-farm transmission of ASFV was considered plausible in the newly affected area (2024), in the area where the 16 outbreaks included in the control cases occurred, and where clustering between outbreaks was described by Bellini et al. [8]. Furthermore, these outbreaks do not appear to be spatially connected to positive wild boars (Figure 2).

## 4. Discussion

In Lombardy, outbreaks of ASF occurred in domestic pigs in both 2023 and 2024, with all outbreaks taking place in commercial pig farms. In fact, pigs on family run farms had been slaughtered before the introduction of ASF in the province of Pavia. The owners received compensation for not reintroducing pigs, and these farms remained empty.

To gain knowledge of the factors involved in the spread of ASFV, a case–control study was conducted on the outbreaks in 2024. There were 21 outbreaks, but five had to be excluded because there were no farms that met the matching criteria for the outbreaks located in the area involved in the epidemic in 2023. Indeed, in this area, pig population density was very low because it remained subject to restrictions due to the presence of ASF in wild boars and the repopulation of pig farms was not permitted. Thus, a total of 48 pigs farms were enrolled in the study: 16 outbreaks and 32 control farms.

The results of the case–control study showed that the presence of a house on the farm premises was strongly associated with increased risk of infection. Other factors positively associated with the risk of ASF included contact by the owner, family members, or employees with other pig farms, the presence of agricultural vehicles inside the clean area of the farm, the use of internal vehicles to transfer manure outside the farm, and visits by external personnel to the clean area of the farm, including those involved in general maintenance. These results seem to confirm the relevance of human activities to the spread of ASFV [9].

All the above-mentioned factors could have been effectively mitigated by adopting strict external biosecurity measures, meaning the set of measures aimed at preventing pathogenic agents from entering or escaping a farm, which also include cleaning and disinfection. One element of risk that emerged in all outbreaks is the location of the disinfection point, which was inside the farm [8]. This is permitted by current legislation but to be effective requires constant and correct application of cleaning and disinfection procedures of all vehicles or tools brought into the farm, including farm vehicles used for agricultural work, which in most cases were housed on the farm.

On the other hand, in line with that mentioned above, the results of the study also showed that measures aimed at strengthening the segregation of the farm from the external environment (external biosecurity), such as the mandatory passage through a hygiene lock upon entry, the presence of a hygiene lock for farm personnel and external visitors, the presence of special equipment and disinfection points at the entrance to the sheds, the loading of dead pigs outside the animal housing area and the ownership of non-adjacent agricultural land, were associated with reduced risk.

The role of swill feeding in the transmission of infection was assessed by the study but was not found to be significant. The explanation for this could be that swill feeding is more commonly practiced on family farms, which had been subject to mandatory slaughter before the outbreak of infection and therefore were not involved in the epidemic. As already mentioned, all the outbreaks occurred on commercial farms, where swill feeding is impractical, at least in the Italian pig farming context.

Movement of infected animals is often associated with the spread of the infection [23], though in the epidemic described in this study, this occurred only once. This can be explained by the fact that, in 2024, most of the outbreaks occurred in areas already regionalized, due to the presence of ASF in wild boar; therefore, pig movements were already restricted.

Regarding the results relating to territorial risk, the first five outbreaks (those excluded from the case–control analysis) were found to be the closest to the positive wild boars, with minimum and median distances of 3.53 km and 9.01 km, respectively. Conversely, for the remaining outbreaks, the minimum and median distances from the positive wild boars were 22.24 km and 28.51 km.

This could be explained by the fact that the first five outbreaks occurred in an area already affected in 2023 and where the disease had continued to spread in wild boars. The remaining outbreaks occurred in a different area where, to date, the disease has never been identified in wild boars and where it is believed that the transmission of the infection occurred between farms [8]. Based on the analyses conducted, these outbreaks do not appear to be linked to the cases detected in wild boars in the same period (Figure 2).

Several studies have been conducted to investigate the role of blood-feeding arthropods or insects as mechanical vectors of ASFV but there is still uncertainty about whether this occurs and if so, to what extent [4]. Despite this, in a case–control study performed in commercial pig farms in Latvia, Poland and Romania, the use of insect nets in windows and openings was identified as a protective factor for ASF outbreaks [4].

In our study, we did not verify the potential effect of mosquito nets as a risk mitigation factor since, on almost all farms in the study area, the pigs were raised indoors, but the boxes had outdoor stalls surrounded by a brick wall, to which the animals had free access. This type of housing makes insect control with mosquito nets unfeasible.

Although the number of farms in the study was limited, the case–control analysis revealed a robust and statistically significant association between the investigated covariates and the introduction of ASFV into pig farms. Nevertheless, the study does have some limitations. The first is the number of farms included, which could have been increased by including the 2023 outbreaks. However, at the time, the infection of pig farms was unexpected, and it was impossible to plan and conduct the case–control studies at the same time as managing the outbreaks. Second, the questionnaire should have been administered simultaneously, on both the control and case farms. This was also infeasible because it would have meant visiting healthy farms in areas where the infection was spreading in both domestic and wild pigs, which is prohibited during epidemics due to the increased risk of transmission of the infection. However, to mitigate the effect of the time lag in the administration of the questionnaires, some of the criteria adopted in farm recruitment were introduced. For example, the questionnaire in the control farms was conducted at the same time as the first biosecurity visit was carried out by local veterinary services at the end of the ASF epidemic in domestic pigs, ensuring that in the meantime there had been no changes in the application of biosecurity.

## 5. Conclusions

Lombardy is an area of intensive pig farming, and the pig sector is of economic relevance for the processing industry, which aims to produce high-quality pork products. However, ASF affected mainly the province of Pavia, one of the province’s least-developed areas for pig farming, and the presence of ASFV in domestic pigs, even only in this area, has had an important socio-economic impact on the entire country.

In Pavia, the disease has been eradicated from domestic pigs, but it continues to be reported in wild boars, which means that it is a continued threat for pig farming. The main risk factors identified in the study appear to be linked to the type of farming in this area and to human activities.

In fact, in this area, pig farming is often combined with other agricultural activities, such as rice and corn cultivation, and, in general, the combination of different agricultural activities within the same facility makes it more difficult to maintain farm biosecurity effectively.

Despite the progress made in research, there is currently no vaccine available against ASF, so the only way to prevent infection is the systematic application of biosecurity, which must be adapted to the specific farming method and local risk factors. Therefore, it is essential to identify the specific risk factors related to farm management in order that mitigation measures can be applied by farmers. Moreover, given the resistance of ASFV, farmers must be made aware of the importance of the correct and systematic application of cleaning and disinfection procedures, which must be considered an integral part of farm biosecurity.

## Figures and Tables

**Table 1:** Conditional logistic regression analysis of risk factors associated with increased likelihood of ASFV infection in pig farms.

Risk Factor	OR	95% CI	*p*-Value
The owner’s, keeper’s, and/or workers’ house is located within the farm (Question n. 2)	20.00	2.56–156.24	0.004
The owner and/or family members and/or employees have relationships with other pig farms (Question n. 3-A)	10.83	1.32–88.70	0.026
Agricultural vehicles (tractors, mixer waggons, trailers, etc.) are kept inside the farm (clean area of the farm) (Question n. 14-A)	4.88	1.03–23.15	0.046
The manure is transferred from the shed to the storage point (slurry tank) through an internal vehicle (Question n. 28)	11.68	1.42–95.92	0.022
In the last month, the clean area of the farm has been visited by internal and external personnel (Question n. 36-B aggregated) *	27.00	1.60–454.21	0.001

* This variable was calculated using McNemar’s correction.

**Table 2:** Conditional logistic regression analysis of biosecurity measures associated with reduced likelihood of ASF infection in pig farms.

Protective Factor	OR	95% CI	*p*-Value
The farm owns other agricultural land that is not contiguous to the main farm (Question n. 13-D)	0.21	0.05–0.77	0.019
A hygiene lock is available for farm personnel and external visitors (Question n. 34-A) *	0.09	0.03–0.311	<0.001
Entrance to the farm is mandatory through a hygiene lock (Question n. 34-B)	0.10	0.02–0.48	0.004
The hygiene lock is fully equipped (Question n. 34-D) *	0.22	0.10–0.50	<0.001
Presence of boot disinfection points at the entrance to the sheds (buckets with disinfectants) (Question n. 35)	0.10	0.01–0.86	0.036
The loading of dead pig carcasses is carried out by the rendering company outside the animal housing and management area (Question n. 39-B)	0.11	0.01–0.93	0.043

* This variable was calculated using McNemar’s correction.

**Table 3:** Descriptive statistics of minimum distance between wild boar and the nearest outbreak (km).

	Outbreaks (No. = 21)	Outbreaks Excluded from the Case–Control Study * (No. = 5)	Outbreaks Included in the Case–Control Study ** (No. = 16)
Minimum	3.53	3.53	22.24
1st quartile	22.24	8.15	24.39
Median	24.70	9.01	28.51
3rd quartile	31.31	11.03	32.39
Maximum	37.49	17.45	37.49

* Outbreaks excluded from the case–control study due to recruitment criteria. ** Outbreaks included in the case–control study due to recruitment criteria.

## Data Availability

Some data presented in this study are available on request due to privacy restrictions.

## Supplementary Materials

The following supporting information can be downloaded at: https://www.mdpi.com/article/10.3390/pathogens15060569/s1, Table S1. The complete questionnaire with questions and sub-questions, for a total of 69 items collecting information on the farm and personnel (30 questions), the presence of wild boar (4 questions), feed, drinking water and bedding (5 questions), farm management practices (5 questions), and biosecurity (25 questions). Table S2. Questionnaire results were summarized using descriptive statistics, reported as frequencies and percentages.
