# Supplementary material for: African Swine Fever (ASF): A Study to Identify Risk Factors Associated with the Introduction of the Disease into Pig Farms
Source: Pathogens. 2026 May 26;15(6):569. doi: 10.3390/pathogens15060569 (PMC13305440; doi:10.3390/pathogens15060569)
Supplement: Supplementary file 1 [file pathogens-15-00569-s001.zip › Bellini_ASF_CaseCcontrolStudy_SM_TableS1.pdf]

Table S1: The complete questionnaire with questions and sub-questions, for a total of 69 items collecting information on the farm and personnel (30 questions), the presence of wild boar (4 questions), feed, drinking water and bedding (5 questions), farm management practices (5 questions), and biosecurity (25 questions).

AFRICAN SWINE FEVER SURVEY

SECTION 1: FARM DETAILS

FARM CODE	
FARM SUBCODE	
TAX ID	
ADDRESS	
CITY	
PROVINCE	
COMPETENT AUTHORITY	
PRODUCTION TYPOLOGY<br>☐ Fattening<br>☐ Reproduction<br>If the type is "Reproduction", indicate the number of sows present:	
NUMBER OF ANIMALS<br>Indicated in BDN:<br>Present at the farm at the time of the visit:	
VISIT DATE	
COMPILER NAME	**Dott.**

SECTION 2: INFORMATION ON THE FARM AND PERSONNEL

		SÌ	NO
1.	How many people work at the farm? ____________<br>Number of owners: ____________<br>Number of employees: ____________		
2.	Is the owner's, keeper's, and/or workers' house located within the farm?	☐	☐
3.	Does the owner and/or family members and/or employees have relationships with other pig farms?<br>If YES, which functions do they carry out:<br>____________<br>____________	☐	☐

4.	Do pigs have access to outdoor areas?	☐	☐

		SÌ	NO
5.	Are there other animal species within the farm?<br>If YES, indicate which ones:<br>☐ Cattle<br>☐ Sheeps<br>☐ Goats<br>☐ Poultry<br>☐ Equines<br>☐ Pets (dogs, cats, etc.)<br>☐ Other species (specify): ____________________	☐	☐
6.	Does the owner have other pig farms?<br>If YES, provide the farm codes of the other farms:<br>____________________<br>____________________<br>____________________<br>____________________<br>____________________<br>If YES, which resources are shared between farms?<br>☐ Personnel<br>☐ Veterinarians<br>☐ Vehicles<br>☐ Feeds<br>☐ Equipment	☐	☐
7.	Does the farm belong to a production chain?	☐	☐
8.	Are employees allowed to bring food for their own consumption into the farm?	☐	☐
9.	Is there a slaughterhouse in the farm?<br>Is there a slaughterhouse of the same ownership in another location?	☐<br>☐	☐<br>☐
10.	Do the owner and/or family members and/or employees also carry out other agricultural activities?<br>If YES, indicate:<br>In the same farm?<br>In other farms (e.g., contractors)?<br>Specify which ones:<br>____________________<br>____________________	☐<br><br>☐<br>☐	☐<br><br>☐<br>☐

11.	Have any new pigs and/or piglets been introduced or purchased on the farm in the last 30 days?	☐	☐
12.	Is there a quarantine period for new introduced animals?	☐	☐

		SÌ	NO
13.	Are there any crops near the farm?	☐	☐
	If YES, indicate which type: ______________________________		
	Are the crops owned by the farm?	☐	☐
	Does the farm own other agricultural land not contiguous to the farm?	☐	☐
14.	Are agricultural vehicles (tractors, mixer wagons, trailers, etc.) kept inside the farm (clean area of the farm)?	☐	☐
	If YES, how many agricultural vehicles are present? __________		
	Are the vehicles inside the farm also used for external agricultural activities?	☐	☐
15.	Is there a documented rodent control plan in place?	☐	☐

SECTION 3: INFORMATION ON WILD BOARS

		SÌ	NO
16.	Have wild boars or other pigs been spotted near the farm (within 100 meters)?	☐	☐
17.	Have any wild boar carcasses or other animal remains been found near the farm (within 100 meters)?	☐	☐
18.	Can wild boars access the farm?	☐	☐
19.	Are there employees who carry out outdoor activities in areas where wild boars are present?	☐	☐

SECTION 4: FEED, DRINKING WATER, BEDDING

		SÌ	NO
20.	Which types of feed are provided to animals on the farm?<br>(*Select all options that correspond to the farm's situation*)<br>☐ Industrial compounds (flours or pellets)<br>☐ Whey<br>☐ Cereals (including self-produced cereals)<br>☐ Hay<br>☐ Fresh grass<br>☐ Products ground or mixed on the farm (farm mill)<br>☐ Others (specify): ____________________		
21.	Are there any signs indicating the feeding of food waste to animals?	☐	☐
22.	Are food waste products fed to pigs?<br>(*To be asked directly to the farmer/operator*)	☐	☐
23.	Which type of water is provided to animals?<br>☐ Well water<br>☐ Rainwater stored in the farm<br>☐ River/lake water<br>☐ Aqueduct/waterwork<br>☐ Others (specify): ____________________		
24.	Which type of bedding is used?<br>☐ Straw<br>☐ Wood chips<br>☐ Sawdust<br>☐ No type of bedding<br>☐ Others (specify): ____________________		

SECTION 5: FARM MANAGEMENT

25. The loading/unloading of animals takes place:

☐ outside of the farm
☐ at the entrance to the sheds
☐ inside the sheds

26. The feed/whey is loaded:

☐ from outside the farm
☐ from inside the farm

27. Slurry loading takes place:

☐ from outside the farm
☐ from inside the farm

28. The manure is transferred from the shed to the storage point (slurry tank) through:

☐ internal vehicle
☐ underground pipes
☐ Others (specify): ______________________________

29. Discard collection takes place:

☐ outside the farm
☐ within the farm
☐ at the entrance to the sheds

SECTION 6: BIOSECURITY

		SÌ	NO
30.	Does the farm have a designated area, located before the entrance barrier, for parking vehicles belonging to farm personnel and/or visitors?	☐	☐
31.	Does the farm have gates or barriers suitable for preventing direct and uncontrolled access by vehicles and/or people?	☐	☐
32.	Is the farm completely fenced in?	☐	☐
33.	Is there a designated area on the premises for disinfection of vehicles?	☐	☐
	Is vehicle access to the farm restricted to passing through this disinfection point?	☐	☐
	Are there drive-through pressure equipment for cleaning, washing, and disinfecting incoming vehicles?	☐	☐
	Are products with proven effectiveness against swine vesicular disease and ASF available and used for vehicle disinfection?	☐	☐
34.	Is there a hygiene lock for farm personnel or external visitors?	☐	☐
	Is the entrance to the farm mandatory through this area?	☐	☐
	Are the sizes of the hygiene lock adequate to the size of the facility (number of employees)?	☐	☐
	Is the hygiene lock equipped?	☐	☐
	☐ Visitors' logbook<br>☐ Boot covers<br>☐ Specific clothes or disposable clothes<br>☐ Disinfectants<br>☐ Working sink<br>☐ Detergent<br>☐ Danish entry<br>☐ Clothes washing area<br>☐ Others (specify): ______________________________		
35.	Are there disinfection points at the entrance to the sheds (buckets with disinfectants)?	☐	☐

ISTITUTO ZOOPROFILATTICO SPERIMENTALE
DELLA LOMBARDIA E DELL'EMILIA ROMAGNA
"BRUNO UBERTINI"
ENTE SANITARIO DI DIRITTO PUBBLICO

		SÌ	NO
36.	Have any visits been carried out in the clean area of the farm in the last month?<br><br>Who has visited the clean area of the farm in the last month?<br>☐ Owners<br>☐ Private veterinarians<br>☐ Official veterinarian<br>☐ Employees (pig farm)<br>☐ Employees (agricultural activities)<br>☐ Consultants<br>☐ Suppliers (feed, bedding, equipment, etc.)<br>☐ Transporters<br>☐ Others (specify): ________________________<br><br>(***Note: Copy of farm logbook or photo!***)	☐	☐
37.	Have there been any worker turnover in the last month?<br>If YES, indicate the type:<br>☐ New hires<br>☐ Temporary replacements (e.g., for vacations)<br>☐ Employment of personnel from other farms<br>☐ Layoffs<br>☐ Others (specify): ___________________________________	☐	☐
	Were the new personnel already adequately trained or experienced in the role?	☐	☐
38.	Have there been any restructuring activities in the farm in the last month?	☐	☐
39.	Are dead animal carcasses removed from the premises within 24 hours from death and stored in a suitable, functioning insulated container or sealed cold storage room located outside the management area, for disposal in accordance with health regulations?	☐	☐
	Is the loading of dead pigs carried out by rendering company outside the animal housing and management area?	☐	☐
	Does the container/cold storage room where the dead animals are kept have a separate access and route from that of the animal housing and management area?	☐	☐
40.	Site of manure collection:<br>☐ Slurry tank<br>☐ Others (specify): _________________________________		
41.	Manure disposal methods:<br>☐ Fertigation in owned fields<br>☐ Fertigation in other affiliated fields		

☐ Biogas
☐ Others (specify): ____________________________________

42. Slurry collection methods:

☐ Farm itself with its own equipment
☐ Other 's vehicles
