# Supplementary material for: African Swine Fever (ASF): A Study to Identify Risk Factors Associated with the Introduction of the Disease into Pig Farms
Source: Pathogens. 2026 May 26;15(6):569. doi: 10.3390/pathogens15060569 (PMC13305440; doi:10.3390/pathogens15060569)
Supplement: Supplementary file 1 [file pathogens-15-00569-s001.zip › Bellini_ASF_CaseCcontrolStudy_SM_TableS2.pdf]

Table S2: Questionnaire results were summarized using descriptive statistics, reported as frequencies and percentages.

SECTION 2: INFORMATION ON THE FARM AND PERSONNEL

- **Question 1**

Question 1-A
How many people work at the farm?

	Cases (n=16)	Controls (n=32)
1	4 (25%)	6 (18.8%)
2	3 (18.8%)	9 (28.1%)
3	2 (12.5%)	5 (15.6%)
4	3 (18.8%)	2 (6.2%)
5	0 (0%)	5 (15.6%)
6	3 (18.8%)	3 (9.4%)
7	1 (6.2%)	1 (3.1%)
10	0 (0%)	1 (3.1%)
Missing	0 (0%)	0 (0%)

Question 1-B
Number of owners

	Cases (n=16)	Controls (n=32)
1	12 (75%)	18 (56.2%)
2	4 (25%)	11 (34.4%)
3	0 (0%)	1 (3.1%)
4	0 (0%)	1 (3.1%)
Missing	0 (0%)	1 (3.1%)

Question 1-C
Number of employees

	Cases (n=16)	Controls (n=32)
0	6 (37.5%)	8 (25%)
1	1 (6.2%)	8 (25%)
2	2 (12.5%)	7 (21.9%)
3	3 (18.8%)	2 (6.2%)
4	1 (6.2%)	5 (15.6%)
5	3 (18.8%)	1 (3.1%)
8	0 (0%)	1 (3.1%)
Missing	0 (0%)	0 (0%)

- **Question 2**
  Is the owner's, keeper's, and/or workers' house located within the farm?

	Cases (n=16)	Controls (n=32)
No	6 (37.5%)	31 (96.9%)
Yes	10 (62.5%)	1 (3.1%)
Missing	0 (0%)	0 (0%)

- **Question 3**

Question 3-A
Does the owner and/or family members and/or employees have relationships with other pig farms?

	Cases (n=16)	Controls (n=32)
No	6 (37.5%)	24 (75%)
Yes	10 (62.5%)	8 (25%)
Missing	0 (0%)	0 (0%)

Question 3-B
If YES, which functions do they carry out:

	Cases (n=16)	Controls (n=32)
114XXXXX, 139XXXXX	1 (6.2%)	0 (0%)
Owner	5 (31.2%)	0 (0%)
Owner (also other species)	1 (6.2%)	0 (0%)
Owner does not enter clean area (ZP): 004XXXXX	0 (0%)	1 (3.1%)
Owner does not enter clean area (ZP): 006XXXXX	0 (0%)	1 (3.1%)
Owner does not enter clean area (ZP): 037XXXXX	0 (0%)	1 (3.1%)
Owner does not enter clean area (ZP): 087XXXXX	0 (0%)	1 (3.1%)
Owner does not enter clean area (ZP): 129XXXXX	0 (0%)	1 (3.1%)
Owner enters clean area (ZP): 151XXXXX	0 (0%)	1 (3.1%)
Owner enters clean area (ZP):158XXXXX	0 (0%)	1 (3.1%)
Two holders of the same farm with four barns	1 (6.2%)	0 (0%)
Veterinarian for several farms belonging to the same owner	1 (6.2%)	0 (0%)
Veterinary employee enters the clean area (ZP) of other farms	0 (0%)	1 (3.1%)
Workers	1 (6.2%)	0 (0%)
Missing	6 (37.5%)	24 (75%)

- **Question 4**
  Do pigs have access to outdoor areas?

	Cases (n=16)	Controls (n=32)
No	16 (100%)	17 (53.1%)
Yes	0 (0%)	15 (46.9%)
Missing	0 (0%)	0 (0%)

- **Question 5**

Question 5-A
Are there other animal species within the farm?

	Cases (n=16)	Controls (n=32)
Cattle, goats, horses, poultry, other species	1 (6.2%)	0 (0%)
Cattle, pets (cat, dog, etc.)	1 (6.2%)	1 (3.1%)
Horses	1 (6.2%)	0 (0%)
Horses, pets (cat, dog, etc.)	1 (6.2%)	0 (0%)
Horses, sheep	1 (6.2%)	0 (0%)
Pets (cat, dog, etc.)	3 (18.8%)	14 (43.8%)
Pets (cat, dog, etc.), other species	0 (0%)	1 (3.1%)
Sheep	1 (6.2%)	0 (0%)
No	7 (43.8%)	16 (50%)
Missing	0 (0%)	0 (0%)

Question 5-B: If YES, indicate which ones

	Cases (n=16)	Controls (n=32)
3	1 (6.2%)	0 (0%)
Cat	0 (0%)	8 (25%)
Cattle farming in the same courtyard	0 (0%)	1 (3.1%)
Cattle in the dirty area	0 (0%)	1 (3.1%)
Dogs, hens in the dirty area near the clean area	0 (0%)	1 (3.1%)
In dirty area, a dairy barn houses 350 lactating cows plus replacement heifers	0 (0%)	1 (3.1%)
Rabbits, llamas	1 (6.2%)	0 (0%)
Missing	14 (87.5%)	20 (62.5%)

- **Question 6**

Question 6-A
Does the owner have other pig farms?

	Cases (n=16)	Controls (n=32)
No	7 (43.8%)	22 (68.8%)
Yes	9 (56.2%)	10 (31.2%)
Missing	0 (0%)	0 (0%)

Question 6-B

If YES, provide the farm codes of the other farms

Code other farms	Cases (n=16)	Controls (n=32)
004XXXXX (complete cycle)	0 (0%)	1 (3.1%)
009XXXXX (farm with no animals)	0 (0%)	1 (3.1%)
006XXXXX (complete cycle)	0 (0%)	1 (3.1%)
037XXXXX (complete cycle)	0 (0%)	1 (3.1%)
050XXXXX (farm with no animals)	0 (0%)	1 (3.1%)
085XXXXX (complete cycle)	0 (0%)	1 (3.1%)
087XXXXX (complete cycle)	0 (0%)	1 (3.1%)
151XXXXX (complete cycle)	0 (0%)	1 (3.1%)
152XXXXX	1 (6.2%)	0 (0%)
158XXXXX (complete cycle)	0 (0%)	1 (3.1%)
160XXXXX	1 (6.2%)	0 (0%)
012XXXXX (complete cycle),<br>129XXXXX (complete cycle)	0 (0%)	1 (3.1%)
114XXXXX,<br>139XXXXX	1 (6.2%)	0 (0%)
004XXXXX,<br>021XXXXX,<br>036XXXXX (outbreak)	1 (6.2%)	0 (0%)
004XXXXX,<br>097XXXXX,<br>181XXXXX	1 (6.2%)	0 (0%)
005XXXXX (farm with no animals),<br>024XXXXX,<br>045XXXXX	1 (6.2%)	0 (0%)
047XXXXX,<br>058XXXXX,<br>125XXXXX	1 (6.2%)	0 (0%)
004XXXXX (pigs),<br>004XXXXX (pigs),<br>021XXXXX (pigs),<br>031XXXXX (pigs),<br>036XXXXX/3 (goats),<br>036XXXXX/4 (chickens),<br>036XXXXX/5 (cattle),<br>036XXXXX/6 (rabbits),<br>036XXXXX/7 (horses),<br>036XXXXX/8 (llamas),<br>045XXXXX (pigs),<br>050XXXXX (pigs),<br>060XXXXX (pigs)	1 (6.2%)	0 (0%)
Closed at ASF onset	1 (6.2%)	0 (0%)
Missing	7 (43.8%)	22 (68.8%)

Question 6-C

If YES, which resources are shared between farms?

	Cases (n=16)	Controls (n=32)
Animals, personnel, vehicles	1 (6.2%)	0 (0%)
Equipment	0 (0%)	3 (9.4%)
Equipment, feeds, personnel, vehicles	1 (6.2%)	0 (0%)
Equipment, personnel	0 (0%)	1 (3.1%)
Equipment, personnel, veterinarians, vehicles	1 (6.2%)	0 (0%)
Feeds, personnel, vehicles	0 (0%)	2 (6.2%)
Feeds, personnel, vehicles, veterinarians	0 (0%)	1 (3.1%)
Personnel	1 (6.2%)	0 (0%)
No shared resources	5 (31.2%)	3 (9.4%)
Missing	7 (43.8%)	22 (68.8%)

- **Question 7**

Does the farm belong to a production chain?

	Cases (n=16)	Controls (n=32)
No	7 (43.8%)	20 (62.5%)
Yes	9 (56.2%)	12 (37.5%)
Missing	0 (0%)	0 (0%)

- **Question 8**

Are employees allowed to bring food for their own consumption into the farm?

	Cases (n=16)	Controls (n=32)
No	15 (93.8%)	30 (93.8%)
Yes	1 (6.2%)	2 (6.2%)
Missing	0 (0%)	0 (0%)

- **Question 9**

Question 9-A

Is there a slaughterhouse in the farm?

	Cases (n=16)	Controls (n=32)
No	15 (93.8%)	31 (96.9%)
Yes	1 (6.2%)	1 (3.1%)
Missing	0 (0%)	0 (0%)

Question 9-B

Is there a slaughterhouse of the same ownership in another location?

	Cases (n=16)	Controls (n=32)
No	16 (100%)	32 (100%)
Yes	0 (0%)	0 (0%)
Missing	0 (0%)	0 (0%)

- **Question 10**

Question 10-A
Do the owner and/or family members and/or employees also carry out other agricultural activities?

	Cases (n=16)	Controls (n=32)
No	8 (50%)	12 (37.5%)
Yes	8 (50%)	20 (62.5%)
Missing	0 (0%)	0 (0%)

Question 10-B
If yes, in the same farm?

	Cases (n=16)	Controls (n=32)
No	2 (12.5%)	2 (6.2%)
Yes	6 (37.5%)	18 (56.2%)
Missing	8 (50%)	12 (37.5%)

Question 10-C
If yes, in other farms (e.g., contractors)?

	Cases (n=16)	Controls (n=32)
No	4 (25%)	16 (50%)
Yes	4 (25%)	4 (12.5%)
Missing	8 (50%)	12 (37.5%)

Question 10-D
If yes, specify which ones

	Cases (n=16)	Controls (n=32)
Biogas, cattle farm, fields	1 (6.2%)	2 (6.2%)
Cattle farm, fields	0 (0%)	4 (12.5%)
Farm of other species	1 (6.2%)	0 (0%)
Farm of other species, fields	1 (6.2%)	0 (0%)
Fields	3 (18.8%)	7 (21.9%)
Fields, slurry spreading	0 (0%)	4 (12.5%)
Other pig farms	0 (0%)	1 (3.1%)
Slurry spreading	0 (0%)	3 (9.4%)
Slurry tank used by another farm	0 (0%)	2 (6.2%)
Missing	10 (62.5%)	9 (28.1%)

- **Question 11**
Have any new pigs and/or piglets been introduced or purchased on the farm in the last 30 days?

	Cases (n=16)	Controls (n=32)
No	14 (87.5%)	22 (68.8%)
Yes	2 (12.5%)	10 (31.2%)
Missing	8 (50%)	0 (0%)

- **Question 12**

  Is there a quarantine period for new introduced animals?

	Cases (n=16)	Controls (n=32)
No	4 (25%)	1 (3.1%)
Yes	2 (12.5%)	9 (28.1%)
Not applicable	10 (62.5%)	22 (68.8%)
Missing	0 (0%)	0 (0%)

- **Question 13**

Question 13-A

Are there any crops near the farm?

	Cases (n=16)	Controls (n=32)
No	2 (12.5%)	1 (3.1%)
Yes	14 (87.5%)	31 (96.9%)
Missing	0 (0%)	0 (0%)

Question 13-B

If YES, indicate which type

	Cases (n=16)	Controls (n=32)
Alfalfa, barley, corn, ryegrass	0 (0%)	1 (3.1%)
Alfalfa, corn, rice,	1 (6.2%)	0 (0%)
Alfalfa, corn, sorghum, wheat	0 (0%)	1 (3.1%)
Alfalfa, poplars, rapeseed, rice	1 (6.2%)	0 (0%)
Barley, rice	1 (6.2%)	0 (0%)
Barley, soy	0 (0%)	1 (3.1%)
Corn	6 (37.5%)	9 (28.1%)
Corn, barley, orchard, soy	0 (0%)	1 (3.1%)
Corn, hay, rice	1 (6.2%)	0 (0%)
Corn, meadows	0 (0%)	6 (18.8%)
Corn, poplars	0 (0%)	1 (3.1%)
Corn, poplars, rice,	0 (0%)	1 (3.1%)
Corn, poplars, soy	0 (0%)	1 (3.1%)
Corn, rice	3 (18.8%)	5 (15.6%)
Corn, sorghum	0 (0%)	1 (3.1%)
Corn, soy	0 (0%)	1 (3.1%)
Corn, sunflower, vineyards	0 (0%)	1 (3.1%)
Corn, triticale	0 (0%)	1 (3.1%)
Rice	1 (6.2%)	0 (0%)
Missing	2 (12.5%)	1 (3.1%)

Question 13-C
Are the crops owned by the farm?

	Cases (n=16)	Controls (n=32)
No	8 (50%)	10 (31.2%)
Yes	6 (37.5%)	21 (65.6%)
Missing	2 (12.5%)	1 (3.1%)

Question 13-D
Does the farm own other agricultural land not contiguous to the farm?

	Cases (n=16)	Controls (n=32)
No	10 (62.5%)	7 (21.9%)
Yes	6 (37.5%)	25 (78.1%)
Missing	0 (0%)	0 (0%)

- **Question 14**

Question 14-A
Are agricultural vehicles (tractors, mixer wagons, trailers, etc.) kept inside the farm (clean area of the farm)?

	Cases (n=16)	Controls (n=32)
No	3 (18.8%)	17 (53.1%)
Yes	13 (81.2%)	15 (46.9%)
Missing	0 (0%)	0 (0%)

Question 14-B
If YES, how many agricultural vehicles are present?

	Cases (n=16)	Controls (n=32)
0	11 (68.8%)	0 (0%)
1 (animal transport vehicle, internal use only – 129XXXXX)	0 (0%)	1 (3.1%)
1 (feed mixer wagon)	0 (0%)	1 (3.1%)
1 (telescopic also used for other farms)	0 (0%)	1 (3.1%)
2	1 (6.2%)	0 (0%)
2 (tractor with tank and tractor with front loader plus mower)	0 (0%)	1 (3.1%)
5–6 (boat, construction machinery and various others)	0 (0%)	1 (3.1%)
2 tractors, feed mixer wagon, harrow, BCS, fertilizer spreader	0 (0%)	2 (6.2%)
12 tractors and wagons	1 (6.2%)	0 (0%)
15–20 (wagons, tractors, 2 tanks, plow, burier, etc.)	0 (0%)	1 (3.1%)
Forklift, cart for moving pigs and disused equipment	0 (0%)	1 (3.1%)
Many assorted equipment	0 (0%)	1 (3.1%)
Plow, trailer, small truck, tank	0 (0%)	1 (3.1%)
Tractor and cart	0 (0%)	1 (3.1%)

Tractor and tank (used only internally, declared not approved)	0 (0%)	1 (3.1%)
Tractors, harrows, telescopic handler, hose for umbilical system	0 (0%)	1 (3.1%)
Trailers, plow, harrow	0 (0%)	1 (3.1%)
Missing	3 (18.8%)	17 (53.1%)

Question 14-C
Are the vehicles inside the farm also used for external agricultural activities?

	Cases (n=16)	Controls (n=32)
No	10 (62.5%)	6 (18.8%)
Yes	4 (25%)	9 (28.1%)
Missing	2 (12.5%)	17 (53.1%)

SECTION 3: INFORMATION ON WILD BOARS

- **Question 15**
  Is there a documented rodent control plan in place?

	Cases (n=16)	Controls (n=32)
No	0 (0%)	1 (3.1%)
Yes	16 (100%)	31 (96.9%)
Missing	0 (0%)	0 (0%)

- **Question 16**
  Have wild boars or other pigs been spotted near the farm (within 100 meters)?

	Cases (n=16)	Controls (n=32)
No	16 (100%)	32 (100%)
Yes	0 (0%)	0 (0%)
Missing	0 (0%)	0 (0%)

- **Question 17**
  Have any wild boar carcasses or other animal remains been found near the farm (within 100 meters)?

	Cases (n=16)	Controls (n=32)
No	16 (100%)	32 (100%)
Yes	0 (0%)	0 (0%)
Missing	0 (0%)	0 (0%)

- **Question 18**
  Can wild boars access the farm?

	Cases (n=16)	Controls (n=32)
No	16 (100%)	30 (93.8%)
Yes	0 (0%)	2 (6.2%)
Missing	0 (0%)	0 (0%)

- **Question 19**
  Are there employees who carry out outdoor activities in areas where wild boars are present?

	Cases (n=16)	Controls (n=32)
No	16 (100%)	32 (100%)
Yes	0 (0%)	0 (0%)
Missing	0 (0%)	0 (0%)

SECTION 4: FEED, DRINKING WATER, BEDDING

- **Question 20**
  Which types of feed are provided to animals on the farm?

	Cases (n=16)	Controls (n=32)
Cereals (including self-produced cereals)	2 (12.5%)	0 (0%)
Cereals (including self-produced cereals), industrial compounds (flours or pellets)	1 (6.2%)	1 (3.1%)
Cereals (including self-produced cereals), industrial compounds (flours or pellets), products ground or mixed on farm (farm mill)	0 (0%)	5 (15.6%)
Cereals (including self-produced cereals), industrial compounds (flours or pellets), products ground or mixed on farm (farm mill)	1 (6.2%)	0 (0%)
Cereals (including self-produced cereals), industrial compounds (flours or pellets), products ground or mixed on farm (farm mill), whey	0 (0%)	4 (12.5%)
Cereals (including self-produced cereals), industrial compounds (flours or pellets), whey	1 (6.2%)	0 (0%)
Cereals (including self-produced cereals), industrial compounds (flours or pellets), whey, other	1 (6.2%)	0 (0%)
Cereals (including self-produced cereals), products ground or mixed on farm (farm mill), whey	0 (0%)	2 (6.2%)
Industrial compounds (flours or pellets)	5 (31.2%)	13 (40.6%)
Industrial compounds (flours or pellets), products ground or mixed on farm (farm mill)	1 (6.2%)	0 (0%)
Industrial compounds (flours or pellets), products ground or mixed on farm (farm mill), whey	0 (0%)	2 (6.2%)
Industrial compounds (flours or pellets), whey	3 (18.8%)	2 (6.2%)
Products ground or mixed on farm (farm mill)	1 (6.2%)	3 (9.4%)
Missing	0 (0%)	0 (0%)

- **Question 21**
  Are there any signs indicating the feeding of food waste to animals?

	Cases (n=16)	Controls (n=32)
No	16 (100%)	32 (100%)
Yes	0 (0%)	0 (0%)
Missing	0 (0%)	0 (0%)

- **Question 22**
  Are food waste products fed to pigs?

	Cases (n=16)	Controls (n=32)
No	16 (100%)	32 (100%)
Yes	0 (0%)	0 (0%)
Missing	0 (0%)	0 (0%)

- **Question 23**
  Which type of water is provided to animals?

	Cases (n=16)	Controls (n=32)
Tap water	6 (37.5%)	2 (6.2%)
Well water	10 (62.5%)	30 (93.8%)
Missing	0 (0%)	0 (0%)

- **Question 24**
  Which type of bedding is used?

	Cases (n=16)	Controls (n=32)
No type of bedding	16 (100%)	31 (96.9%)
Straw	0 (0%)	1 (3.1%)
Missing	0 (0%)	0 (0%)

SECTION 5: FARM MANAGEMENT

- **Question 25**
  The loading/unloading of animals takes place.

	Cases (n=16)	Controls (n=32)
At the entrance to the sheds	12 (75%)	26 (81.2%)
Inside the sheds	0 (0%)	0 (0%)
Outside of the farm	4 (25%)	6 (18.8%)
Missing	0 (0%)	0 (0%)

- **Question 26**
  The feed/whey is loaded:

	Cases (n=16)	Controls (n=32)
From inside the farm	12 (75%)	20 (62.5%)
From outside the farm	4 (25%)	12 (37.5%)
Missing	0 (0%)	0 (0%)

- **Question 27**
  Slurry loading takes place:

	Cases (n=16)	Controls (n=32)
From inside the farm	5 (31.2%)	4 (12.5%)
From outside the farm	11 (68.8%)	28 (87.5%)
Missing	0 (0%)	0 (0%)

- **Question 28**
  The manure is transferred from the shed to the storage point (slurry tank) through:

	Cases (n=16)	Controls (n=32)
Internal vehicle	8 (50%)	4 (12.5%)
Underground pipes	8 (50%)	28 (87.5%)
Missing	0 (0%)	0 (0%)

- **Question 29**
  Discard collection takes place:

	Cases (n=16)	Controls (n=32)
At the entrance to the sheds	0 (0%)	2 (6.2%)
Outside the farm	11 (68.8%)	25 (78.1%)
Within the farm	5 (31.2%)	5 (15.6%)
Missing	0 (0%)	0 (0%)

SECTION 6: BIOSECURITY

- **Question 30**
  Does the farm have a designated area, located before the entrance barrier, for parking vehicles belonging to farm personnel and/or visitors?

	Cases (n=16)	Controls (n=32)
No	2 (12.5%)	0 (0%)
Yes	14 (87.5%)	32 (100%)
Missing	0 (0%)	0 (0%)

- **Question 31**
  Does the farm have gates or barriers suitable for preventing direct and uncontrolled access by vehicles and/or people?

	Cases (n=16)	Controls (n=32)
No	4 (25%)	3 (9.4%)
Yes	12 (75%)	29 (90.6%)
Missing	0 (0%)	0 (0%)

- **Question 32**
  Is the farm completely fenced in?

	Cases (n=16)	Controls (n=32)
No	5 (31.2%)	5 (15.6%)
Yes	11 (68.8%)	27 (84.4%)
Missing	0 (0%)	0 (0%)

- **Question 33**

Question 33-A
Is there a designated area on the premises for disinfection of vehicles?

	Cases (n=16)	Controls (n=32)
No	0 (0%)	0 (0%)
Yes	16 (100%)	32 (100%)
Missing	0 (0%)	0 (0%)

Question 33-B
Is vehicle access to the farm restricted to passing through this disinfection point?

	Cases (n=16)	Controls (n=32)
No	6 (37.5%)	4 (12.5%)
Yes	10 (62.5%)	28 (87.5%)
Missing	0 (0%)	0 (0%)

Question 33-C
Are there drive-through pressure equipment for cleaning, washing, and disinfecting incoming vehicles?

	Cases (n=16)	Controls (n=32)
No	0 (0%)	0 (0%)
Yes	16 (100%)	32 (100%)
Missing	0 (0%)	0 (0%)

Question 33-D
Are products with proven effectiveness against swine vesicular disease and ASF available and used for vehicle disinfection?

	Cases (n=16)	Controls (n=32)
No	0 (0%)	0 (0%)
Yes	16 (100%)	32 (100%)
Missing	0 (0%)	0 (0%)

- **Question 34**

Question 34-A
Is there a hygiene lock for farm personnel or external visitors?

	Cases (n=16)	Controls (n=32)
No	3 (18.8%)	0 (0%)
Yes	13 (81.2%)	32 (100%)
Missing	0 (0%)	0 (0%)

Question 34-B
Is the entrance to the farm mandatory through this area?

	Cases (n=16)	Controls (n=32)
No	11 (68.8%)	4 (12.5%)
Yes	5 (31.2%)	28 (87.5%)
Missing	0 (0%)	0 (0%)

Question 34-C
Are the sizes of the hygiene lock adequate to the size of the facility (number of employees)?

	Cases (n=16)	Controls (n=32)
No	4 (25%)	2 (6.2%)
Yes	12 (75%)	30 (93.8%)
Missing	0 (0%)	0 (0%)

Question 34-D
Is the hygiene lock equipped?

	Cases (n=16)	Controls (n=32)
No	7 (43.8%)	0 (0%)
Yes	9 (56.2%)	32 (100%)
Missing	0 (0%)	0 (0%)

Question 34-E
What is the filter area equipped with?

	Cases (n=16)	Controls (n=32)
Boot covers, clothes washing area, Danish entry, detergent, disinfectants, specific or disposable clothes, visitors' logbook, working sink	8 (50%)	1 (3.1%)
Boot covers, clothes washing area, Danish entry, detergent, disinfectants, specific or disposable clothes, working sink	0 (0%)	1 (3.1%)
Boot covers, clothes washing area, Danish entry, detergent, disinfectants, specific or disposable clothes, working sink, other	0 (0%)	1 (3.1%)
Boot covers, Danish entry, detergent, disinfectants, specific or disposable clothes, visitors' logbook, working sink	0 (0%)	12 (37.5%)
Boot covers, Danish entry, detergent, disinfectants, specific or disposable clothes, working sink	0 (0%)	7 (21.9%)
Boot covers, Danish entry, detergent, specific or disposable clothes, visitors' logbook, working sink	0 (0%)	2 (6.2%)
Boot covers, Danish entry, detergent, specific or disposable clothes, visitors' logbook, working sink, other	0 (0%)	1 (3.1%)
Boot covers, Danish entry, detergent, specific or disposable clothes, working sink	0 (0%)	2 (6.2%)
Boot covers, Danish entry, disinfectants, specific or disposable clothes, visitors' logbook, working sink	0 (0%)	1 (3.1%)
Boot covers, Danish entry, disinfectants, specific or disposable clothes, working sink	0 (0%)	1 (3.1%)
Boot covers, Danish entry, specific or disposable clothes	1 (6.2%)	0 (0%)
Boot covers, Danish entry, specific or disposable clothes, visitors' logbook, working sink	0 (0%)	2 (6.2%)
Boot covers, detergent, disinfectants, specific or disposable clothes, working sink	0 (0%)	1 (3.1%)
Boot covers, detergent, specific or disposable clothes, working sink	1 (6.2%)	0 (0%)
Missing	6 (37.5%)	0 (0%)

Question 34-F
Specify if ‘other’

	Cases (n=16)	Controls (n=32)
Filter area set up at the entrance to the sheds at the moment	1 (6.2%)	0 (0%)

Non-through shower	0 (0%)	1 (3.1%)
Shower	0 (0%)	1 (3.1%)
Missing	15 (93.8%)	30 (93.8%)

- **Question 35**
  Are there disinfection points at the entrance to the sheds (buckets with disinfectants)?

	Cases (n=16)	Controls (n=32)
No	5 (31.2%)	1 (3.1%)
Yes	11 (68.8%)	31 (96.9%)
Missing	0 (0%)	0 (0%)

- **Question 36**

Question 36-A
Have any visits been carried out in the clean area of the farm in the last month?

	Cases (n=16)	Controls (n=32)
No	0 (0%)	1 (3.1%)
Yes	16 (100%)	31 (96.9%)
Missing	0 (0%)	0 (0%)

Question 36-B
Who has visited the clean area of the farm in the last month?

	Cases (n=16)	Controls (n=32)
Consultants, employees (agricultural activities), employees (pig farm), official veterinarian, owners, private veterinarians	1 (6.2%)	0 (0%)
Consultants, employees (agricultural activities), employees (pig farm), official veterinarian, owners, private veterinarians, suppliers, transporters	0 (0%)	1 (3.1%)
Consultants, employees (pig farm), maintenance technician, official veterinarian, owners, private veterinarians, transporters	1 (6.2%)	0 (0%)
Consultants, employees (pig farm), official veterinarian, owners, photovoltaic panel workers (electricians, maintenance workers entering with external opening machines (gate)), private veterinarians	1 (6.2%)	0 (0%)
Consultants, official veterinarian	1 (6.2%)	0 (0%)
Consultants, official veterinarian, owners	0 (0%)	1 (3.1%)
Consultants, owners, private veterinarians	1 (6.2%)	0 (0%)
Employees (agricultural activities), employees (pig farm), fence workers, official veterinarian, owners, private veterinarians	1 (6.2%)	0 (0%)

Employees (agricultural activities), employees (pig farm), official veterinarian, owners	0 (0%)	1 (3.1%)
Employees (agricultural activities), employees (pig farm), official veterinarian, owners, suppliers	0 (0%)	1 (3.1%)
Employees (agricultural activities), employees (pig farm), owners, transporters	1 (6.2%)	0 (0%)
Employees (pig farm)	0 (0%)	1 (3.1%)
Employees (pig farm), official veterinarian, owners	0 (0%)	6 (18.8%)
Employees (pig farm), official veterinarian, owners, private veterinarians	2 (12.5%)	0 (0%)
Employees (pig farm), official veterinarian, owners, private veterinarians, Consortium	1 (6.2%)	0 (0%)
Employees (pig farm), official veterinarian, owners, private veterinarians, suppliers, transporters	0 (0%)	2 (6.2%)
Employees (pig farm), official veterinarian, owners, suppliers	0 (0%)	2 (6.2%)
Employees (pig farm), official veterinarian, owners, suppliers, transporters	0 (0%)	3 (9.4%)
Employees (pig farm), official veterinarian, owners, transporters	1 (6.2%)	1 (3.1%)
Employees (pig farm), owners, private veterinarians	0 (0%)	2 (6.2%)
Employees (pig farm), owners, suppliers	0 (0%)	1 (3.1%)
Employees (pig farm), owners, suppliers, transporters	0 (0%)	1 (3.1%)
Official veterinarian, owners	1 (6.2%)	2 (6.2%)
Official veterinarian, owners, private veterinarians	3 (18.8%)	0 (0%)
Official veterinarian, owners, private veterinarians, suppliers, transporters	0 (0%)	1 (3.1%)
Official veterinarian, owners, suppliers	0 (0%)	1 (3.1%)
Official veterinarian, owners, suppliers, transporters	0 (0%)	2 (6.2%)
Owners	1 (6.2%)	2 (6.2%)
Missing	0 (0%)	1 (3.1%)

Question 36-B *aggregated*

	**Cases (n=16)**	**Controls (n=32)**
Internal personnel	13 (81.2%)	32 (100%)
Internal and external personnel	3 (18.8%)	0 (0%)
Missing	0 (0%)	0 (0%)

- **Question 37**

Question 37-A
Have there been any worker turnover in the last month?

	Cases (n=16)	Controls (n=32)
No	15 (93.8%)	31 (96.9%)
Yes	1 (6.2%)	1 (3.1%)
Missing	0 (0%)	0 (0%)

Question 37-B
If YES, indicate the type

	Cases (n=16)	Controls (n=32)
New hires	0 (0%)	1 (3.1%)
Temporary replacements	1 (6.2%)	0 (0%)
Employment of personnel from other farms	0 (0%)	0 (0%)
Layoffs	0 (0%)	0 (0%)
Missing	15 (93.8%)	31 (96.9%)

Question 37-C
Were the new personnel already adequately trained or experienced in the role?

	Cases (n=16)	Controls (n=32)
No	1 (6.2%)	0 (0%)
Yes	0 (0%)	1 (3.1%)
Missing	15 (93.8%)	31 (96.9%)

- **Question 38**
Have there been any restructuring activities in the farm in the last month?

	Cases (n=16)	Controls (n=32)
No	13 (81.2%)	30 (93.8%)
Yes	3 (18.8%)	2 (6.2%)
Missing	0 (0%)	0 (0%)

- **Question 39**

Question 39-A
Are dead animal carcasses removed from the premises within 24 hours from death and stored in a suitable, functioning insulated container or sealed cold storage room located outside the management area, for disposal in accordance with health regulations?

	Cases (n=16)	Controls (n=32)
No	1 (6.2%)	3 (9.4%)
Yes	15 (93.8%)	29 (90.6%)
Missing	0 (0%)	0 (0%)

Question 39-B

Is the loading of dead pigs carried out by rendering company outside the animal housing and management area?

	Cases (n=16)	Controls (n=32)
No	6 (37.5%)	3 (9.4%)
Yes	10 (62.5%)	29 (90.6%)
Missing	0 (0%)	0 (0%)

Question 39-C
Does the container/cold storage room where the dead animals are kept have a separate access and route from that of the animal housing and management area?

	Cases (n=16)	Controls (n=32)
No	4 (25%)	5 (15.6%)
Yes	12 (75%)	27 (84.4%)
Missing	0 (0%)	0 (0%)

- **Question 40**
Site of manure collection:

	Cases (n=16)	Controls (n=32)
Slurry tank	16 (100%)	32 (100%)
Missing	0 (0%)	0 (0%)

- **Question 41**
Manure disposal methods:

	Cases (n=16)	Controls (n=32)
Biogas	8 (50%)	5 (15.6%)
Biogas, fertigation in owned fields	0 (0%)	11 (34.4%)
Fertigation in other affiliated fields	5 (31.2%)	0 (0%)
Fertigation in owned fields	2 (12.5%)	8 (25%)
Fertigation in owned fields, fertigation in other affiliated fields	0 (0%)	7 (21.9%)
Fertigation in owned fields, fertigation in other affiliated fields, biogas	0 (0%)	1 (3.1%)
Missing	1 (6.2%)	0 (0%)

- **Question 42**
Slurry collection methods:

	Cases (n=16)	Controls (n=32)
Farm itself with its own equipment	8 (50%)	25 (78.1%)
Other 's vehicles	8 (50%)	7 (21.9%)
Missing	0 (0%)	0 (0%)
